# The impact of electronic and conventional cigarettes on periodontal health—a systematic review and meta-analysis

**DOI:** 10.1007/s00784-023-05162-4

**Published:** 2023-08-01

**Authors:** Daniel G. E. Thiem, Phil Donkiewicz, Raha Rejaey, Nadine Wiesmann-Imilowski, James Deschner, Bilal Al-Nawas, Peer W. Kämmerer

**Affiliations:** 1 Department of Oral and Maxillofacial Surgery, Facial Plastic Surgery, University Medical Center Mainz, Augustusplatz 2, 55131 Mainz, Germany; 2 Department of Periodontology and Operative Dentistry, University Medical Center Mainz, Augustusplatz 2, 55131 Mainz, Germany; 3 BioHorizons Camlog, Maybachstr. 5, 71299 Wimsheim, Germany

**Keywords:** Electronic cigarettes, Smoke, Oral health, Periodontal health, Vaporizer, Smoking, Bleeding on probing

## Abstract

**Objectives:**

This systematic review and meta-analysis examined the effects of electronic cigarettes on periodontal health compared to conventional cigarette smoke and a non-smoking population.

**Materials and methods:**

MEDLINE, Embase, Web of Science, CENTRAL, and ClinicalTrials.gov were screened for literature. Eligibility criteria included clinical studies published between 2006 and 2022 that compare e-cigarettes and conventional cigarettes on periodontal health (bleeding on probing (BoP), plaque index (PI), probing depth (PD), attachment loss (AL), marginal bone loss (MBL), tooth loss, molecular inflammation markers, salivary flow rate). Meta-regression analysis was used to examine the influence of moderator variables.

**Results:**

Sixteen studies were found to be eligible for qualitative synthesis. Individual analyses showed that cigarette smokers had significantly higher PI, PD, AL, and MBL and increased concentrations of proinflammatory mediators than e-cigarette users and non-smokers. Meta-analysis revealed a 0.33-fold lower chance for BoP in e-cigarette users compared to smokers (*p* = 0.03), whereby meta-regression failed to detect any effects regarding the age of users and frequency of smoking. A 0.01-fold decreased chance for positive BoP in e-cigarette users compared with non-smokers was seen (*p* < 0.01).

**Conclusions:**

The current findings suggest that that e-cigarette use might be considered a healthier alternative to cigarette smoking concerning periodontal health. Even so, harmful effects of electronic nicotine delivery system (ENDS) usage on periodontal health were seen as well. However, a definitive decision on this research question remains elusive due to the absence of randomized controlled trials.

**Clinical relevance:**

Electronic cigarettes, marketed as a safer alternative to traditional cigarettes, are becoming increasingly popular. Evidence on the use of electronic cigarettes as a cessation aid and its beneficial impact compared to cigarette smoke remains inconclusive, so the analysis conducted in this review addresses a recent question of high clinical relevance.

## Introduction

According to the World Health Organization (WHO) forecast, the number of smokers in the German population over 15 years old could reach around 16.2 million by 2025. In the USA, on average, about 1500 youth aged 12–17 smoke their first cigarette daily, and more than 200 adolescents aged 12–17 become daily smokers. This would continue the downward trend, and the number would be more than a quarter lower than in 2000 (22.22 million). Regular smoking can cause various pathologies such as cardiovascular disease [1], respiratory disorders [2], and periodontitis [3–6] and is the single most preventable cause of death worldwide. Triggers for many pathologies include over 90 proven carcinogenic and toxic cigarette substances, some resulting from the burn process. These include polycyclic hydrocarbons, nitrosamines, and aldehydes [7, 8]. Electronic nicotine delivery systems (ENDS) (e.g., electronic cigarettes, vaporizers, vape pens, shisha pens, and e-pipes) are said to prevent the formation of unwanted products by bypassing the combustion process.

The tobacco industry and related industries market and promote ENDS as “safer” alternatives to traditional cigarettes, and many users consider them to be significantly “less harmful” than tobacco products, particularly cigarettes [9]. Consequently, the rising frequency of use of electronic cigarettes, particularly in the USA and Europe, with prevalence rates of regular and/or current use among adults ranging between 0.9 and 1.8%, respectively, is unsurprising [10–12]. By 2018, more than 3.6 million adolescents had tried ENDS, including 4.9% of middle school students and 20.8% of high school students [13]. Initial scientific studies reporting lower physical harm caused by ENDS and emphasizing their benefits in smoking cessation further promoted their popularity and international sales.

While Public Health England and the Royal College of Physicians argued for a 95% reduction in health risks from electronic cigarettes based on evidence from short-term studies [14, 15], another set of experimental studies, such as those from the University of Rochester’s School of Medicine and Dentistry, refuted the harmlessness of vaporizers [16]. To date, evidence on using ENDS as a cessation aid is inconclusive. Since the oral cavity, the first upper respiratory tract station, is the primary exposed region when smoke is introduced, the influence on oral health and here specially on periodontal health is significant. Current studies proved that smoking and vaping are risk factors (ENDS: odds ratio = 2.3, 95% confidence interval (CI) = 1.52 to 3.59; conventional cigarettes: odds ratio = 2.2, 95% CI = 1.76 to 2.68) for periodontal disease [17] with about eight million of periodontal disease cases in Germany and about forty million worldwide linked to smoking [18]. One of the main reasons is smoke-related functional and morphological impairment of gingival fibroblasts [19]. Besides, smokers have been reported to have a poorer oral hygiene when compared to non-smokers [20]; tar in tobacco products might conduct pigmentation and accumulation of bacteria on tooth surfaces [21]. Nicotine-dependent oral effects are local vasoconstriction and a reduced blood flow that will reduce gingival oxygen and blood supply. Tobacco and ENDS might also decrease oral immunoglobulin levels [22] and alter the oral microbiome [23], leading to several pathogenic microbes [21, 24]. Smoking results in discoloration of the tooth structure; changes in taste and olfactory perception are also reported [5]. In addition to periodontal disease, cigarette smoke is considered a significant cause in the development of oral squamous cell carcinoma [25–27]. This systematic review and meta-analysis aimed to determine whether and to what extent the consumption of ENDS bears advantages and disadvantages on periodontal health (bleeding on probing (BoP), plaque index (PI), probing depth (PD), attachment loss (AL), marginal bone loss (MBL), tooth loss, molecular inflammation markers, salivary flow rate) compared to conventional cigarette smoke and non-smokers.

## Materials and methods

The present meta-analysis was performed based on the recommendations and principles of the Preferred Reporting Items for Systematic Reviews and Meta-Analysis Statement (PRISMA) [28–30]. The *Cochrane Reviewers’ Handbook* was used as a resource for this review. The inclusion and exclusion criteria were set according to the patient, intervention, comparator, outcome, and studies (PICOS) model [31].

### Focused question

For the present review, the focused PI(CO) question to be addressed was as follows: “To what extent does oral health differ between e-cigarette users, cigarette smokers, or non-smokers?”

- “Population”: e-cigarette users, smokers, and non-smokers
- “Intervention”: clinical inspection of the oral mucosa, radiographic imaging, and histological assessment
- “Comparison”: e-cigarette users, smokers, and non-smokers
- “Outcome”: bleeding on probing (BoP), plaque index (PI), probing depth, attachment loss, marginal bone loss (MBL), tooth loss, molecular inflammation markers, an salivary flow rate

### Search strategy

To identify relevant studies, a systematic electronic search was conducted using the terms adjusted according to the pattern developed for MEDLINE (OVID): “oral health AND (electronic cigarette OR electronic nicotine delivery system OR vaporizer)”. Multiple synonyms were included in the literature search to encompass as many publications as possible. Search terms had “oral health,” “electronic cigarette,” “electronic nicotine delivery system,” and “Vaporizer”. The authors searched for relevant studies in MEDLINE (OVID), Embase (OVID; 2006–04/2022), Web of Science, CENTRAL (The Cochrane Library, 2022), and ClinicalTrials.gov. Articles in German and English were screened. Based on the fact that publications with relatively high effect sizes are more likely to be published than studies with smaller effect sizes and the former are more likely to be respected in the meta-analysis, it should be taken into account that any bias can be articulated as publication bias in the literature and also in the meta-analysis [32].

### Meta-regression

Meta-regression allows examination of the influence of the so-called moderator variables or influencing variables on the effect size [32, 33]. Since the number of studies eligible for meta-analysis is usually limited, there is a great risk of overfitting; hence, only a few explanatory variables were chosen in this meta-regression.

Meta-regressions were used to test the influence of different moderators (age, duration of exposure, frequency of exposure, time of cessation, and dropout rates) on pooled estimates. In the case of substantial heterogeneity between the studies, the statistician has to explore possible causes, which can be done by covariates on the study level that could explain the differences between the studies. After testing each variable in the model, residual heterogeneity (*I*^2^) and the amount of heterogeneity accounted for each variable (*R*^2^) were calculated. A funnel plot was established to detect publication bias, whereby a symmetrical distribution of studies in the plot indicates a low risk [32]. Over-publication of studies is one potential cause for the risk of publication bias [32]. The trim-and-fill analysis was applied for bias correction, an iterative process that verifies the number of missing publications necessary for a symmetrical result in the funnel plot. The algorithm is terminated when symmetry is present, and a “corrected” effect size is obtained [32, 33].

### Eligibility criteria

The inclusion criteria were as follows: (a) studies published between January 2006 and April 2022, (b) all studies that compare the clinical effect of e-cigarettes to conventional cigarettes on oral health, namely, periodontal health. The primary examination parameter was bleeding on probing (BoP). The bleeding index is a widely established diagnostic procedure [34] and indicates clinical [35–40], histological [41–43], and bacterial [44–47] alterations of the gingiva [48]. In the literature, the BoP is presented as an objective and simple method which, in contrast to purely visual diagnostics, is used for practical dichotomous early detection of gingivitis [48]. The correct technique is performed using a probe applying a sounding pressure of approximately 0.25 Newton and checking the gingiva positively or negatively (after 10–15 s) for bleeding on probing [49]. Secondary examination parameters included plaque index (PI), probing depth (PD), attachment loss (AL), marginal bone loss (MBL), tooth loss, molecular inflammation markers, and salivary flow rate. No selection based on other clinical, histologic, or radiographic examination methods as well as age, gender, or social origin was conducted. Studies that did not compare inhalation products were excluded, as studies without a control group other than e-cigarette users (healthy non-smokers or cigarette smokers).

### Data extraction

The following items were extracted from publications that met the inclusion criteria: author, year, country, study design, sample size, measures of exposure (smoking status), measures of outcome (BoP, PI, PD, AL, MBL, tooth loss, molecular inflammation markers, salivary flow rate), results, conclusions, conflict of interest, and source of funding.

### Study selection

The duplicate check was performed according to the standardized procedure based on the Bramer method. Steps 1 and 2 were performed automatically (duplicates can be declared as such). Steps 3–6 included intellectual screening (Table 1) [50]. To avoid bias in study selection, abstract screening was performed by two independent reviewers (PWK and RR). Discrepancies were discussed afterward and evaluated by a third independent reviewer (DGET).

**Table 1:** Duplicate check approach

Step	Process	Validation
1. Author|year|title|secondary title (journal)	Automated	Pages
2. Author|year|title|pages	Automated	Secondary title–volume–pages
3. Title|volume|pages	Intellectual	Author and year
4. Author|volume|pages	Intellectual	Title
5. Year|volume|issue|pages	Intellectual	Author–title
6. Title	Intellectual	Author–year–pages
7. Author|year	Intellectual	Title

### Quality assessment

Due to the growing number of studies, it is essential to summarize publications on a specific topic, critically evaluate them, and design a science-based recommendation towards clinical practice [51]. Specific exclusion criteria were established during the literature search to guarantee the review’s validity to exclude irrelevant data. Studies were individually pooled, and an effect measure was determined for each. The effect measure of individual studies was then formulated at the review level as the overall effect of the intervention. The means and standard deviations calculated in the individual studies were used to merge different scales or rankings. Instead of providing the standardized mean difference as an effect size, we converted it to the odds ratio and their respective 95% confidence intervals (CIs) [32]. Heterogeneity was tested using Cochran’s *Q* test and quantified using the *I*-square test (level of inconsistency) and Tau^2^ (estimate of between-study variance). The risk of bias in cohort studies was assessed using a modified version of the Newcastle–Ottawa scale (NOS) [52]. According to the description, however, the scale refers to cohort and case–control studies. Nevertheless, other observational studies, such as cross-sectional, were assigned to the two subgroups and assessed. To assess the selected studies’ quality of evidence and the quality classification for validity control, the GRADE (Grading of Recommendations Assessment, Development, and Evaluation) approach, which focuses on evaluating the study design, was performed [53].

Data analyses of longitudinal studies were performed using the statics software R (R Studio, Version 1.0.143, R Foundation for Statistical Computing, Vienna, Austria. ISBN 3–900051-07–0, URL: http://www.R-project.org/).

## Results

### Search results and excluded publications

**Fig. 1:**
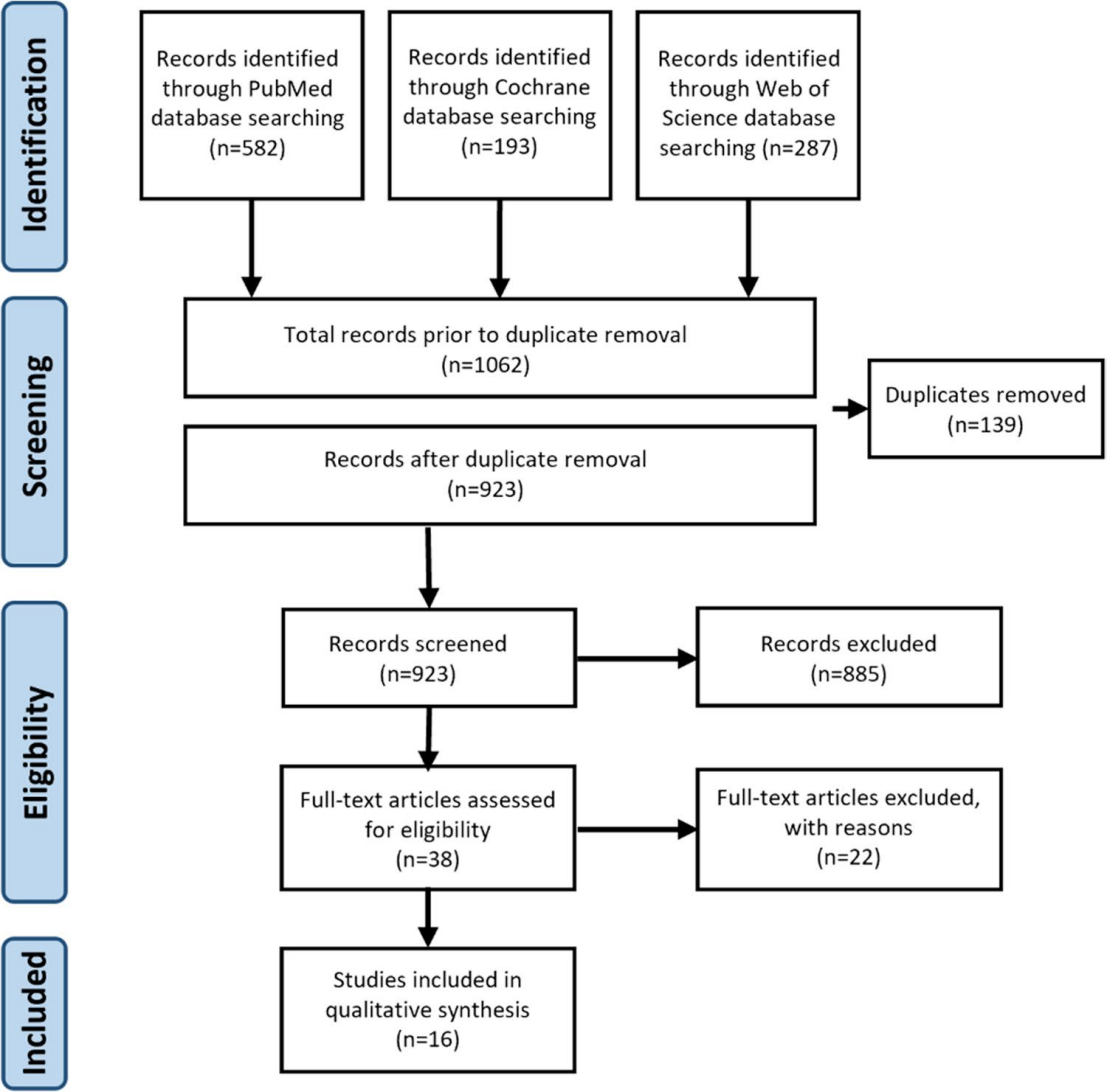
Flowchart of studies screened, retrieved, included, and analyzed in the systematic review and subsequent meta-analyses according to the PRISMA guidelines [28, 30]

From a total of 923 publications identified, 885 studies were excluded after a review of titles and abstracts. In the next step, 38 articles were evaluated based on the complete text, with another exclusion of 22 articles due to a lack of comparison of electronic and conventional cigarette users and the need for control groups. Since the systematic review aimed to investigate a clinical correlation, experimental-only studies were excluded, leaving 16 studies eligible for the qualitative synthesis of this review [20, 54–65]. A flowchart diagram depicts the screening process (Fig. 1).

### Summary of the included studies

- Evidence level of included studies
  The following table lists all publications that met the inclusion criteria (Table 2). We applied the GRADE classification of evidence to evaluate the study concept/study design (Table 3). Since all studies were non-randomized, they can only be classified as evidence grade III or IIa.
- Quality assessment of included studies
  In this section, validity-controlled studies are listed according to their quality class. Thereby, certain criteria with different prioritization were defined for evaluating the articles. The weighting of the criteria is based on the probability and the size of possible biases. Since the sources of bias are more significant in the methodological application of a study (randomization, investigation of established hypotheses), these criteria were weighted twice (Table 4). Clustering was generally based on methodology assessment and dental and professional realization (measurement tools, patient population, results). Benchmarks were selected to allow examination of the studies for objectivity, reliability, and validity and to provide universality of criteria to enable comparison of the articles. In addition, the publishing journal was included as part of the quality assessment of the studies. The peer review process is based on the study assessment by independent scientists of reputable journals and positively influenced the scoring. The individual heading explanation can be found in Table 5. The item “objective comparison of substances of consumption” addresses uniform conditions among the population. Thus, patients who use e-cigarettes for 6 months are compared with those who smoked cigarettes for 1 year. Ultimately, all scores are summed up, resulting in an ordinal scale of the respective studies. The evaluation was carried out by two reviewers independently and was then compared (Table 6).
- Studies included according to the Newcastle–Ottawa scale
  In this type of evaluation, three main categories were differentiated: selection, comparability, and exposure/outcome. Each category contains about three to four questions, which the reviewer had to answer and evaluate separately for each study. Only for comparability, two stars can be given. The score total resulted in an ordinal classification of the studies (Tables 7 and 8).

**Table 2:** Included studies listed by level of evidence and year of publication

Nr	Author	Title	Level of evidence
1	[54]	The association between electronic-cigarette use and self-reported oral symptoms including cracked or broken teeth and tongue and/or inside-cheek pain among adolescents: a cross-sectional study	III
2	[55]	Oral mucosal lesions in electronic cigarettes consumers versus former smokers	III
3	[56]	Comparison of periodontal parameters and self-perceived oral symptoms among cigarette-smokers, individuals vaping electronic cigarettes and never-smokers: a pilot study	IIa
4	[57]	Oral *Candida* carriage among cigarette- and waterpipe- smokers, and electronic cigarette users	IIa
5	[58]	Association of e-cigarette use with oral health: a population based cross-sectional questionnaire study?	III
6	[59]	Electronic cigarette: role in the primary prevention of oral cavity cancer	IIa
7	[57]	Clinical and radiographic periodontal status and whole salivary cotinine, IL1β and IL-6 levels in cigarette- and waterpipe-smokers and e-cig users	IIa
8	[61]	Clinical periodontal status and gingival crevicular fluid cytokine profile among cigarette-smokers, electronic-cigarette users and never-smokers	IIa
9	[62]	Deregulation of biologically significant genes and associated molecular pathways in the oral epithelium of electronic cigarette users	IIa
10	[63]	Impact of cigarette smoking and vaping on the outcome of full-mouth ultrasonic scaling among patients with gingival inflammation: a prospective study	IIa
11	[64]	Association between regular electronic nicotine product use and self-reported periodontal disease status: population assessment of tobacco and health survey	III
12	[65]	Oral health of smokers and e-cigarette users: a case–control study	IIa
13	[20]	Proinflammatory cytokine levels and peri-implant parameters among cigarette smokers, individuals vaping electronic cigarettes and non-smokers	IIb
14	[66]	Comparison of self-rated oral symptoms and periodontal status among cigarette smokers and individuals using electronic nicotine delivery systems	III
15	[67]	Comparison of cotinine levels in the peri-implant sulcular fluid among cigarette and waterpipe smokers, electronic-cigarette users, and non-smokers	III
16	[68]	Comparison of RANKL and osteoprotegerin levels in the gingival crevicular fluid of young cigarette- and waterpipe-smokers and individuals using electronic nicotine delivery systems	III

**Table 3:** Evidence grade classification according to GRADE [53]

Level of evidence	Type of study
Ia	Evidence based on meta-analyses of randomized controlled trials in systematic reviews
Ib	Evidence based on at least one randomized controlled trial
IIa	Evidence based on at least one well-designed controlled trial without randomization
IIb	Evidence based on at least one well-designed, quasi-experimental study
III	Evidence based on well-designed, nonexperimental cohort studies
IV	Expert opinion

**Table 4:** Explanation of quality classification point assignment

Symbol	Meaning
+	Meeting the criterion, simple scoring
+ +	Meets the criterion, double rating
−	Does not meet criterion

**Table 5:** Annotation of the quality classification modified according to Shabazfar et al. [69]

Scale	Criterion	Meaning of criterion	Score
Journal	“Peer review”	Increased result validity through peer review	Simple
Study design	Randomization	Random allocation of the subjects?	Double
	Investigation of established hypotheses	Have hypotheses been made and answered?	Double
Measurement tools	Suitable measuring instrument	Objectifiable measurements?	Simple
Patients	Objective comparison of two consumables	Possible comparison of subjects?	Simple
	Number	Sufficient patient number? (> 35)	Simple
Results	Adequate level of data aggregated	Have results been compiled appropriately?	Simple
	Investigator	Number of investigators mentioned? How many?	Simple

**Table 6:** Quality classification of the included studies based on objective and comparable criteria with *=patient survey, **=only one tooth per sextant examined, and +=two investigators

Study	Journal	Study design		Measurement tools	Patients		Results		Grade
	Peer review	Randomization	Investigation of established hypotheses	Suitable measuring instrument	Objective comparison of two consumables	Group size > 35	Adequate level of data aggregated	Investigator	
[54]	+	−	−	− *	+	+	+	−	4
[55]	+	−	−	+	+	+	+	+ +	6
[56]	+	−	+ +	+	+	−	+	+	7
[57]	+	−	+ +	+	−	−	+	+	6
[58]	+	−	+ +	− *	+	+	+	−	6
[59]	+	−	−	+	+	−	+	+ +	5
[57]	+	−	+ +	+	+	+	+	+	8
[61]	+	−	−	+	+	+	+	+	6
[62]	+	−	−	+	−	−	+	−	3
[20]	+	−	+ +	+	+	−	+	+	7
[63]	+	−	+ +	+	+	−	+	+	7
[64]	+	−	+ +	− *	+	+	+	−	6
[65]	+	−	−	− **	+	+	+	+	5
[20]	+	−	−	+	+	−	+	+	5
[66]	+	−	−	+	+	−	+	+	5
[67]	+	−	+ +	+	+	−	+	+	7
[68]	+	−	−	+	+	−	+	+	5

**Table 7:** Assessment of case–control studies using the Newcastle–Ottawa scale

Study	Selection				Comparability	Exposure			∑
	Question				Question	Questions			
	(1)	(2)	(3)	(4)	(1)	(1)	(2)	(3)	
[55]	*				*	*		*	4
[65]	*				*	*	*	*	5
[58]			*		*	(*)	*		4
[56]		*	*	*	*	*	*		6
[63]		*	*	*	*	*	*	*	7
[20]		*	*	*	*	*	*		6
[68]		*	*	*	*	*	*	*	7
[66]		*	*	*	*	*	*		6

**Table 8:** Assessment of cohort studies using the Newcastle–Ottawa scale

Study	Selection				Comparability	Outcome			∑
	Question				Question	Questions			
	(1)	(2)	(3)	(4)	(1)	(1)	(2)	(3)	
[60]		*	*	*	*	*	*		6
[64]		*	*	*	*		*		5
[54]		*	*	*	*				4
[57]		*	*	*	*	*	*		6
[59]			*	*	*	*	*		5
[61]		*	*	*	*	*	*		6
[62]		*	*	*	*	*	*		6
[67]		*	*	*	*	*	*	*	7

### Descriptive comparison of the studies

#### Periodontal parameters for the classification of oral health

- Gingival bleeding/bleeding on probing
  Varying periodontal parameters were used to evaluate oral health in e-cigarette users, cigarette smokers, and non-smokers in the included studies. In addition, the findings of the studies were collected in different ways, i.e., using different methods. While results derived from subjective reports by study participants were utilized in some studies [54, 64], others conducted clinical assessments [20, 56, 57, 61, 63, 65]. For instance, Cho demonstrated that both non-smokers and e-cigarette users had an equal or similar odds ratio concerning gingival pain and bleeding [54], whereas Atuegwu et al. demonstrated that e-cigarette users had an increased tendency to gingival disease (OR 1.76; 95% CI 1.12–2.76) [64]. Similar results were highlighted by Ghazali et al. using a so-called gingival bleeding index which was significantly increased (*p* = 0.001) in e-cigarette users compared with non-smokers [65]. In the same study, according to Löe and Silness, the gingival index was also assessed and was highest in smokers, followed by non-smokers and e-cigarette users. The difference between cigarette smokers and non-smokers (*p* = 0.001) as well as between smokers and e-cigarette users (*p* = 0.000) was significant in each case [65]. Five studies analyzed bleeding on probing (BoP) [20, 56, 57, 61, 63], with all articles showing significantly increased sulcular bleeding in non-smokers compared with the other groups. The differences were significant with *p* < 0.05 [57, 61] and *p* < 0.01 [20, 56, 63], respectively. While four publications reported smokers with the second highest BoP, followed by e-cigarette users [20, 56, 57, 63], BinShabaib et al. identified the second highest BoP in cigarette smokers [61]. However, all of the studies highlighted the slight difference between e-cigarette users and cigarette smokers and classified the two products as having similar adverse effects on oral health.
- Plaque index (PI)
  Plaque index as another periodontal parameter was shown to be increased in cigarette smokers compared to e-cigarette users and non-smokers in six of the included studies [20, 56, 57, 61, 63, 65]. Thereby, the differences were found significant in all studies (*p* < 0.05 [57, 61]; *p* < 0.01 [20, 56, 65]) except for that by ALHarthi et al. [63]. However, the authors found a significantly increased index at the three- and 6-month follow-up after periodontal therapy in smokers compared with e-cigarette users and non-smokers (*p* < 0.05), with no difference between the latter two groups. In contrast, the study by ArRejaie et al. showed a significantly increased plaque index in e-cigarette users compared with non-smokers [20]. At the same time, no differences were found in the remaining studies in this regard [56, 61, 65].
- Probing depth (PD)
  Probing depth was addressed in five studies listed above [20, 56, 57, 61, 63]. This revealed a significant increase in probing depth in smokers compared with the other two groups of e-cigarette users and non-smokers, each with *p* < 0.05 [57, 61] and *p* < 0.01 [20, 56], respectively. No difference was found between non-smokers and e-cigarette users.
- Attachment loss (AL)
  Regarding attachment loss, two studies reported significantly increased (*p* < 0.05) values in cigarette smokers compared with the other two groups [57, 61]. In contrast, Javed et al. and ALHarthi et al. found no differences between the groups [56, 63].
- Marginal bone loss (MBL)
  Marginal bone loss was included in assessing oral health in five studies [20, 56, 57, 61, 64]. Cigarette smokers were found to have a significantly (*p* < 0.01 [20, 61], *p* < 0.05 [57]) increased mesial and distal bone loss compared to non-smokers, with Javed et al. reporting a significant difference between all groups [56]. Concerning e-cigarette users, two studies showed no difference in bone loss compared with non-smokers [57, 61], whereas two other studies showed significantly increased bone loss among e-cigarette users (OR 1.67; 95% CI 1.06–2.63 [64]; *p* < 0.01 [20]).
- Tooth loss
  Three [56, 61, 63] articles analyzed the number of lost teeth, with cigarette smokers ranking first, followed by e-cigarette users [56, 61] and non-smokers [63], whereby no significance was found.
- Molecular inflammation markers
  Some studies have also investigated molecular inflammation markers within the sulcular fluid. All of the studies found that concentrations of interleukin-1β significantly increased in smokers compared with non-smokers (*p* < 0.05 [61]; *p* < 0.001 [20]; *p* < 0.01 [57]) and e-cigarette users (*p* < 0.01 [20, 57], *p* < 0.05 [61]). ArRejaie et al. further found interleukin-1β levels significantly (*p* < 0.001) increased in e-cigarette users compared with non-smokers [20]. The level of proinflammatory interleukin-6 was enhanced considerably in cigarette smokers compared with non-smokers (*p* < 0.01 [57]) and e-cigarette users (*p* < 0.05 [61]). Similarly, the proinflammatory markers interferon-γ, tumor necrosis factor α, and matrix metalloproteinase MMP-8 (*p* < 0.05 [61]) and MMP-9 (*p* < 0.001 [20]) were significantly increased in smokers compared with non-smokers. In turn, the concentration of MMP-9 was significantly higher in smokers compared with e-cigarette users (*p* < 0.01), as well as higher in e-cigarette users compared with non-smokers (*p* < 0.01) [20].
- Salivary flow rate (SFR)
  Unstimulated total salivary flow rate (UWSFR) and total salivary cotinine did not differ between groups [57]. In this context, ArRejaie et al. quantified the volume of peri-implant sulcular fluid (PISF), which was significantly higher in cigarette smokers and vaping individuals than in non-smokers (*p* < 0.01) [20].

### Pooled outcomes for meta-analysis

- Subgroup analysis of studies with the same study parameters

In most studies, the presence of gingival bleeding was recorded. However, in contrast to the inclusion requirements in this meta-analysis, BoP tests only sometimes assess this. Some studies referred to symptoms from patient reports or other clinical non-standardized bleeding indices [65]. Consequently, only those studies were included for meta-analysis in which means and standard deviations could be identified. Although the standardized mean difference would qualify as a possible effect size, it was converted into the odds ratio for the meta-analysis [32].

### Evaluation of bleeding on probing

**Fig. 2:**
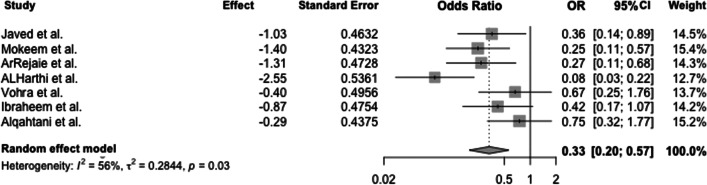
Forest plot for meta-analysis of BoP in e-cigarette users compared to cigarette smokers in three case–control, three cross-sectional, and one cohort studies (*n* = 7 studies, association measure: odds ratio, CI confidence interval)

**Fig. 3:**
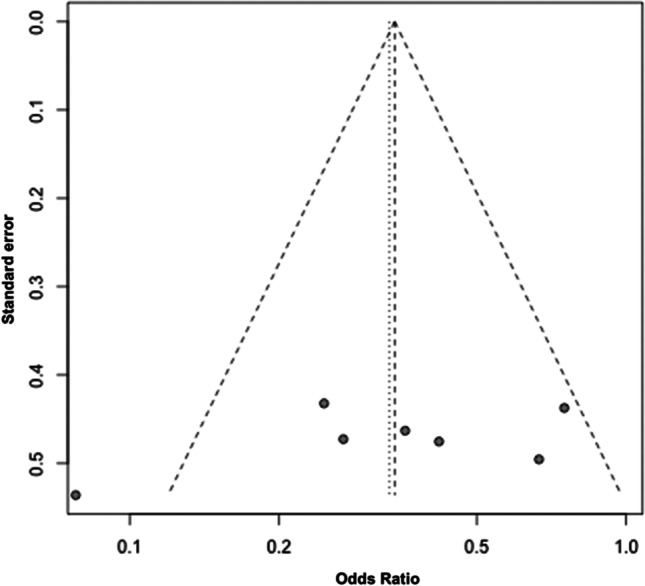
Funnel plot for included studies in this meta-analysis. Each plotted point represents the standard error and standardized odds ratio between e-cigarette users and smokers

- E-cigarette versus cigarette users
  The forest plot shows the odds ratio between e-cigarette users and cigarette smokers for gingival bleeding (positive BoP). A random effects model revealed heterogeneity between the studies (*Q*(6) = 13.7; *p* = 0.03) with *I*^2^ = 56% and *τ*^2^ = 0.28, showing a pooled odds ratio of 0.33. Thus, the odds of a positive BoP are 0.33-fold lower in e-cigarette users than in cigarette smokers (*p* = 0.03) (Fig. 2).
  Furthermore, it was checked whether publication bias was present. Based on the funnel plot, no apparent asymmetry could be detected, so the trim-and-fill algorithm was used to check whether the simulated inclusion of studies would be helpful (Fig. 3). Since the algorithm added no studies, publication bias can be widely excluded.
  The effect of any influencing variables on the pooled odds ratio was also determined. Simple meta-regressions were performed in each case. The following variables were included in the regression analysis: duration and frequency of smoking and age of consumers (Table 9).
  Meta-regression revealed that the age of cigarette smokers does not affect the pooled effect size (*β* =  − 0.02; *p* = 0.79). Therefore, a higher age does not increase the odds ratio, indicating equal chances of positive and negative BoP among e-cigarette users and cigarette smokers. Likewise, there was no effect of the duration of use of conventional cigarettes (*β* =  − 0.03; *p* = 0.64) or e-cigarettes on the pooled effect size (*β* =  − 0.04, *p* = 0.83). Moreover, neither daily e-cigarette use (*β* =  − 0.04; *p* = 0.09) nor everyday use of cigarettes (*β* =  − 0.22, *p* = 0.04) affected the pooled effect size. The chance of bleeding (positive BoP) is equal between e-cigarette smokers and smokers in the case of increased consumption. The remaining influencing variables and moderator variables were not significant.

**Table 9:** Meta-regression investigating the variables influencing the odds ratio for positive BoP between smokers and e-cigarette users

	Model 1		Model 2		Model 3		Model 4		Model 5		Model 6	
Moderator	$\beta$	*p*	$\beta$	*p*	$\beta$	*p*	$\beta$	*p*	$\beta$	*p*	$\beta$	*p*
Axis section	−0.37	0.89	−1.91	0.34	−0.761	0.33	−0.96	0.17	−1.30	0.43	−1.62	≤ 0.001
Age of cigarette smokers	−0.02	0.79										
Age of e-cigarette smokers			0.02	0.68								
Duration of cigarette consumption					−0.03	0.64						
Duration of e-cigarette consumption							−0.04	0.83				
Daily cigarette consumption									0.01	0.96		
Daily e-cigarette consumption											−0.04	0.09
*Q*_Heterogeneity_	13.36	0.02	13.29	0.02	13.01	0.02	13.5	0.02	11.56	0.02	10.9	0.05
*Q*_moderator_	0.07	0.79	0.16	0.68	0.22	0.64	0.05	0.83	0.00	0.96	2.84	0.09
*I*^2^	0.64		0.63		0.63		0.64		0.66		0.53	
${\tau }^{2}$	0.39		0.38		0.37		0.39		0.44		0.00	
*R*^2^	−		−		−		−		−		−

(b) E-cigarette users versus non-smokers

**Fig. 4:**
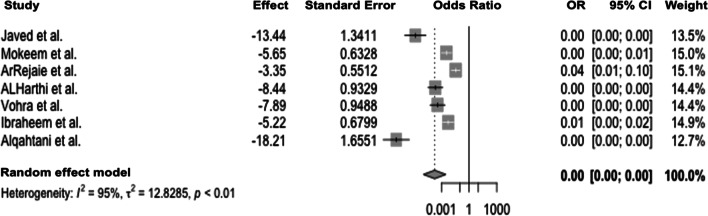
Forest plot for meta-analysis of BoP in e-cigarette users compared to non-smokers in three case–control, three cross-sectional, and one cohort studies (*n* = 7 studies, association measure: odds ratio, CI confidence interval)

**Fig. 5:**
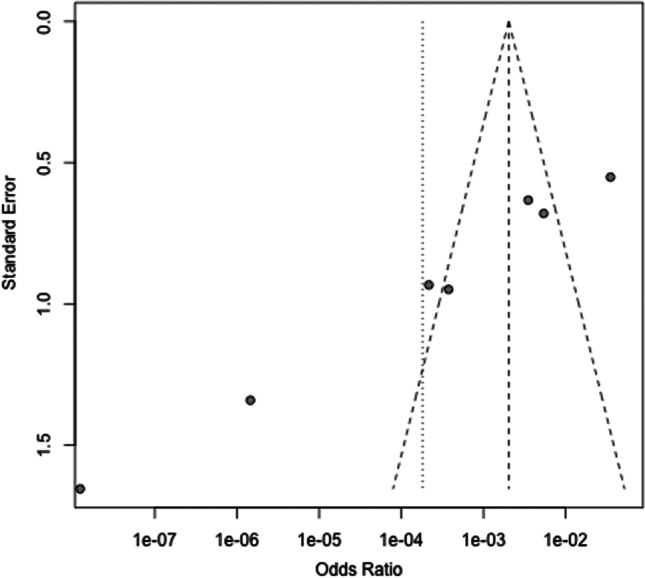
Funnel plot of the meta-analysis of published studies. Each plotted point represents the standard error and standardized odds ratio between e-cigarette users and non-smokers

When comparing e-cigarette users and non-smokers, significant heterogeneity between studies was evident (*Q*(6) = 120.3; *p* < 0.0001), leading to the application of the random effects model with *I*^2^ = 95% and *τ*^2^ = 12.8. The pooled odds ratio of 0.00 (Fig. 4) indicates that e-cigarette users have a lower chance for positive BoP than non-smokers (*p* < 0.01). When assessing for publication bias, effect sizes were distributed asymmetrically in the funnel plot, suggesting publication bias (Fig. 5).

**Fig. 6:**
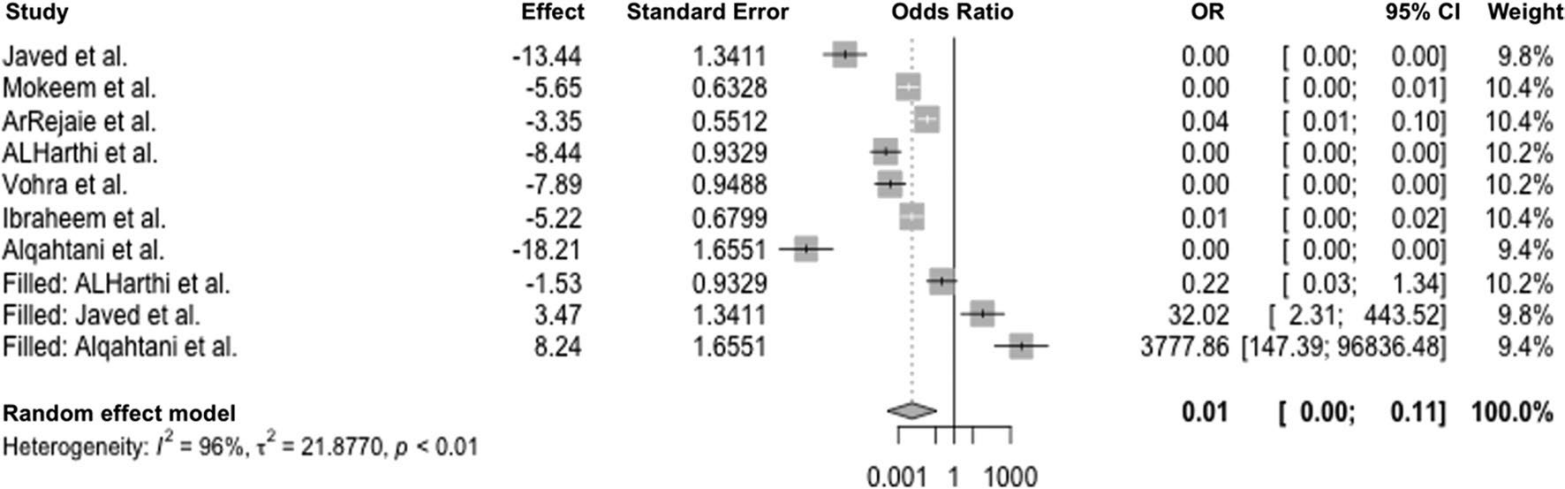
Forest plot illustrating the odds ratio for a positive BoP test comparing e-cigarette users and non-smokers after applying the trim-and-fill method (CI confidence interval, OR odds ratio)

To counteract the publication bias, the trim-and-fill method was applied to estimate the number of additional studies required to minimize the effect of bias and to achieve a symmetric distribution. The following forest plot is augmented with studies according to the above methodology. As a result, significant heterogeneity occurred (*Q*(6) = 71.07; *p* < 0.001). To quantify the heterogeneity, *I*^2^ = 96% and *τ*^2^ = 21.87 were calculated, indicating the presence of considerable heterogeneity. The random effects model yielded a pooled effect size of 0.01, resulting in a 0.01-fold decreased chance of a positive BoP result in e-cigarette users compared with non-smokers (*p* < 0.01; Fig. 6).

Meta-regression analysis revealed no significant results, suggesting that the influence of e-cigarette use has no impact on the relative risk of a positive BoP event compared with non-smokers (Table 10).

**Table 10:** Meta-regression investigating the variables influencing the odds ratio between e-cigarette users and non-smokers

	Model 1		Model 2		Model 3		Model 4	
Moderator	$\beta$	*p*	$\beta$	*p*	$\beta$	*p*	$\beta$	*p*
Axis section	−30.85	0.03	−12.34	0.39	−8.49	0.05	−12.06	0.01
Age of non-smokers	0.58	0.11						
Age of e-cigarette smokers			0.10	0.80				
Daily e-cigarette consumption					−0.02	0.94		
Duration of e-cigarette consumption							0.98	0.43
*Q*_Heterogeneity_	71.01	< 0.0001	117.98	< 0.0001	110.29	< 0.0001	97.92	< 0.0001
*Q*_moderator_	2.48	0.11	0.06	0.80	0.00	0.94	0.63	0.43
*I*^2^	0.97		0.97		0.98		0.97	
${\tau }^{2}$	19.59		29.84		30.13		26.48	
*R*^2^	20.7		0.00		0.00		0.00	

## Discussion

Available data on the oral harms of e-cigarettes remain limited and show little evidence, not least because of the need for high-quality randomized controlled trials. Another problem is the need for comparability of most studies due to individually different, non-standardized compositions of the e-liquids and the vast difference in ENDS systems. While the effects of nicotine on oral mucosal tissue types are known in many aspects, the influence of the regularly added flavor components [70], as well as the carrier substances propylene glycol (PG) and glycerol, especially after vaporization, is mainly unknown [71]. For instance, it has been shown that increased concentrations of menthol are more likely to cause oral mucosal irritation than increased concentrations of nicotine [70]. Commonly reported oral symptoms of e-cigarette use or direct e-liquid exposure included dryness, burning, irritation, bad taste, bad breath, pain, and discomfort [72]. Most of the symptoms were short-term effects that were less frequent or less severe in e-cigarette users than in cigarette smokers.

This systematic review and meta-analysis examined how e-cigarettes and cigarettes affect periodontal health when compared to non-smokers. To objectify the periodontal health status, the parameters: bleeding on probing (BoP), plaque index (PI), periodontal probing depth (PD), attachment loss (AL), marginal bone loss (MBL), and molecular inflammation markers as signs of periodontal inflammation, were systematically summarized. These parameters were regularly analyzed in the literature [20, 72, 73]. Other surrogate parameters were tooth loss rate and unstimulated salivary flow rate.

BoP was the primary test parameter in this study. In clinical practice, BoP is an early marker for gingivitis and periodontal disease [74]. BoP was examined in seven of the included studies, whereby analysis revealed that BoP was significantly increased in non-smokers compared with smokers [20, 56, 57, 61, 63]. Reasons given for the lower bleeding in smokers relate to the vasoconstrictive effects of nicotine [75, 76]. Nonetheless, there is controversy among authors regarding the vasoconstrictive effects, as experimental studies indicated a short-lasting increase in blood flow on nicotine [77–81]. The long-term negative impact of nicotine consumption on gingival blood flow was demonstrated in a study by significantly lower gingival oxygen saturation in smokers compared to non-smokers [82]. Smoking does not seem to influence the microcirculatory vessel quantity, so instead of reducing the number of vessels, a restructuring could be detected [83], which could ultimately promote functional vascular changes causing endothelial dysfunction [84, 85].

Regarding BoP in periodontal screening, it should continually be assessed considering the reduced gingival blood flow in long-term smokers and e-cigarette users [66, 86]. When comparing cigarette smokers and e-cigarette users, there was a 0.33-fold reduced chance of positive BoP in e-cigarette users (*p* = 0.03) [20, 56, 57, 63, 66, 67, 68]. Critically, none of the included studies gave information on the nicotine concentration or the vaporizer models used, which complicates the comparability of the studies. The main problems are associated with different parameters such as coil voltage [88], puff topography [89], and nicotine delivery rates [90]; also, one needs to consider that a majority of e-cigarette users might have smoked conventional cigarettes before and this effect might be an additional bias. Meta-regression analyses examining the influence of various covariates revealed that age did not positively influence the odds of a positive BoP test in cigarette smokers or e-cigarette users. This contradicts the literature and the postulation of decreasing gingival perfusion with increased age [91–94]. Likewise, neither the duration nor the daily use of cigarettes or e-cigarettes significantly impacted a positive BoP event. When comparing non-smokers and e-cigarette users, it was found that the risk of a positive BoP test result was significantly (*p* < 0.01) increased (OR = 0.01) in non-smokers than in e-cigarette users. In addition to the long-term nicotine-induced reduction in gingival perfusion, patient-related (better oral hygiene in e-cigarette users than in non-smokers [20]) or examiner-dependent aspects (varying sounding pressure can lead to false-positive or false-negative results [95] or undetected minor bleeding) may also be influencing factors. Regression analyses in this meta-analysis revealed no significant effect of the covariates age, duration, and frequency of exposure on the risk of bleeding when comparing e-cigarette users and non-smokers.

Regarding the PI, the analyzed studies showed that cigarette smokers presented an increased tendency to form adherent biofilms compared to e-cigarette users and non-smokers [20, 56, 57, 61, 63, 65, 66]. This follows previous findings by Rad et al. and others who demonstrated a significantly (*p* = 0.002) increased PI in cigarette smokers compared with non-smokers [96–99]. Changes in the mineral content due to smoking, namely, a higher calcium concentration in the saliva that could promote plaque accumulation, were initially speculated to be the reason for this observation [100]. However, emission spectrometric analysis refuted this, showing no difference in potassium, sodium, calcium, phosphate, and magnesium in the saliva due to smoking [101]. Instead, smokers showed increased salivary mucosity, which is assumed to result from a smoking-associated alteration of the parotid gland [102].

Similarly, the sublingual and submandibular salivary glands may be affected by smoke in a way to produce predominantly mucosal saliva. Alteration of salivary composition, including enzymes and immunoglobulins, leads to loss of defense functions and may promote plaque formation [102–104]. The influence of the salivary flow rate in this context has been rebutted in the study by Mokeem et al. [57], whereas others demonstrated a significant reduction of salivary flow rate in smokers compared to non-smokers [96, 105]. Possible reasons suggest a chemical-thermal degradation of nitric oxide [106], an autoregulator of salivary secretion, and a reduction of salivary secretion due to nicotine-mediated vasoconstriction [107, 108]. Inadequate oral hygiene [109], which manifests itself in shorter brushing times [110–112], is another aspect that should also be considered for increased plaque formation. Motivation and oral hygiene among e-cigarette users [20] and non-smokers [106, 113, 114] are mostly higher than in smokers.

PD is an important indicator of periodontal health. As the distance between the enamel-cement interface and the sulcus floor increases, the damage to the periodontal attachment area increases as well. Across the reviewed studies in this work, significantly increased probing depth was seen in cigarette smokers compared to e-cigarette users and non-smokers [20, 56, 57, 61, 63]. The suspected cause is nicotine, which induces cell membrane damage, tissue degeneration, endothelial cell damage, and vascular muscle changes in a concentration-dependent manner [3]. The more significant damage in terms of increased probing depths in cigarette smokers compared with e-cigarette users is most likely due to the prolonged exposure to noxious substances in usually older cigarette smokers (mean = 42 years) compared with e-cigarette users (mean = 28 years) [56, 57, 61, 63, 66]. Only one study concluded that PD in e-cigarette users did not significantly differ from smokers. This could be explained using prolonged abuse, i.e., the period during which the user smokes or vapes, and a long smoking history among e-cigarette users [20].

In contrast, other researchers found that e-cigarette users were almost three times more likely to report gingival disease than non-smokers/non-users [115]. This is consistent with the results of the included studies [57, 61, 63]. Pathogenetically, it is assumed that epithelialization, collagen synthesis, and angiogenesis are postponed while immune cell function is reduced at an increased age [91–94, 116]. Clinical attachment loss, defined as the distance between the enamel-cement interface and the gingival margin, was addressed in four included studies [56, 57, 61, 63]. Significantly increased attachment loss was observed in cigarette smokers compared to e-cigarette users and non-smokers [57, 61]. However, all included studies found no difference between e-cigarette users and non-smokers. In brief, one possible explanation could be the younger average age of e-cigarette users with shorter exposition history and superior regeneration potential.

MBL in millimeters, defined as the distance 2 mm below the enamel-cement interface or implant platform to the crestal alveolar bone [20], was analyzed radiographically in all included studies [56, 57, 61]. The *κ*-values for determining the interrater reliability ranged between 0.8 and 0.9. Bone loss was significantly higher in cigarette smokers than in non-smokers in the included studies. In addition to nicotine [117], other noxious substances such as benzenes and cadmium are considered responsible for inhibiting osteoblast proliferation and a chemokine-mediated reduction in bone-forming processes [118–122]. Other impairments from cigarette smoking were also shown concerning vitamin metabolism, as smokers were 50% more likely to have vitamin D deficiency than non-smokers [123]. In combination with a dysregulated calcium metabolism, this could be a possible explanation for an increased MBL in smokers. Compared with e-cigarette users, MBL was also significantly increased in cigarette smokers within the included studies of this meta-analysis [20, 57, 61]. Comparing non-smokers and e-cigarette users, the included literature was inconsistent, ranging from no difference [57, 61] to significantly increased rates of marginal bone loss in e-cigarette users [20].

Concerning the number of lost teeth, the included studies revealed no significant differences between smokers, e-cigarette users, and non-smokers [56, 61, 63]. Potential reasons could involve selecting participants in different age groups and, thus, divergent exposition profiles.

Bioanalytical methods were used to determine molecular markers. For this purpose, either sulcus fluids [20, 61] or saliva samples [57] were taken from the patients. Immunoassays then reveal concentrations of interleukins (IL), interferons (IFN), matrix metalloproteinases (MMP), and tumor necrosis factors (TNF). The included publications indicated significantly increased levels of IL-1β, IL-6, IFN-γ, TNF-α, MMP-8, and MMP-9 in smokers compared with non-smokers [20, 57, 61]. A specific metabolite of nicotine, the alkaloid nornicotine, is assumed to trigger a higher concentration of cytokines. Pathogenetically, nornicotine-triggered overexpression is thought to involve gingival localized receptors for advanced glycation end product (RAGE) expression. RAGE binds proteins and/or lipids, which are glycolyzed into advanced glycation end products (AGEs) after exposure to sugar, causing the release of oxygen radicals and cytokines [124–127]. This results in an excessive immune response, which leads to periodontal degeneration in terms of connective tissue degeneration and osteoclast-mediated bone resorption [126]. As smokers in the included studies had significantly increased cytokine levels, it is questioned whether the formation of the alkaloid nornicotine is reduced or completely absent in e-cigarette users. This was disproved by Bustamante et al. when a transformation product of nornicotine, N′nitrosonornicotine (NNN), was also detected in the urine of e-cigarette users. However, the concentration was significantly reduced in e-cigarette users, which could explain the lower cytokine levels compared to cigarette smokers [128]. While two of the included studies did not find significant mediator differences between e-cigarette users and non-smokers [57, 61], ArRejaie et al. showed significantly increased IL-1β and TNF-α levels in e-cigarette users [20]. Likewise, it is essential to highlight the correlation between elevated mediator levels and increased marginal bone atrophy in cigarette smokers (MMP-9 and IL-1β) and e-cigarette users (IL-1β) [20]. However, both mediators play a crucial role in bone degradation, which nicotine enhances. Accordingly, the observations in the literature are consistent with the findings in the publications included. Smokers demonstrated the highest bone atrophy and levels of MMP-9 and IL-1β compared to e-cigarette users and non-smokers. E-cigarette users also had significantly increased IL-1β and MMP-9 in peri-implant sulcus fluid compared with non-smokers [20]. However, MBL was not increased. This could be explained by the reduced formation of oxygen radicals in e-cigarette users, which has already been demonstrated in vitro [128].

## Conclusion

Based on the present results, it can be summarized that e-cigarette use might be considered a healthier alternative to cigarette smoking concerning periodontal health. Even so, harmful effects of ENDS usage on periodontal health were seen as well. Due to the lack of standardization among studies and randomized controlled trials, a conclusion on the research question remains difficult.

## Data Availability

The datasets used and/or analyzed during the current study are available from the corresponding author upon reasonable request.
